# Sensitive MnFe_2_O_4_–Ag hybrid nanoparticles with photothermal and magnetothermal properties for hyperthermia applications

**DOI:** 10.1039/d1ra03216j

**Published:** 2021-09-08

**Authors:** T. T. N. Nha, P. H. Nam, N. X. Phuc, V. Q. Nguyen, N. H. Nam, D. H. Manh, L. T. Tam, N. T. N. Linh, B. T. V. Khanh, L. T. Lu, L. H. Nguyen, P. T. Phong

**Affiliations:** Graduate University of Science and Technology, Vietnam Academy of Science and Technology 18 Hoang Quoc Viet Hanoi Vietnam; Institute of Materials Science, Vietnam Academy of Science and Technology 18 Hoang Quoc Viet Hanoi Vietnam namph.ims@gmail.com; Duy Tan University 03 Quang Trung Da Nang Vietnam; University of Science and Technology of Hanoi (USTH), Vietnam Academy of Science and Technology 18 Hoang Quoc Viet, Cau Giay Hanoi Vietnam; Vinh University 182 Le Duan Vinh Vietnam; Thai Nguyen University of Sciences Tan Thanh Ward Thai Nguyen City Vietnam; Faculty of Biology, VNU University of Science, Vietnam National University Viet Nam; Institute for Tropical Technology, Vietnam Academy of Science and Technology 18 Hoang Quoc Viet Hanoi Viet Nam; Laboratory of Magnetism and Magnetic Materials, Advanced Institute of Materials Science, Ton Duc Thang University Ho Chi Minh City Vietnam; Faculty of Applied Sciences, Ton Duc Thang University Ho Chi Minh City Vietnam; University of Management and Technology Ho Chi Minh City Vietnam pham.phong@umt.edu.vn

## Abstract

In this study, we present an experiment showing that designing multifunctional MnFe_2_O_4_–Ag nanoparticles to act as a dual hyperthermia agent is an efficient route for enhancing their heating ability. Interestingly, the specific absorption rate of the heteromeric MnFe_2_O_4_–Ag nanoparticles increased 2.7 times under simultaneous irradiation of a 100 Oe magnetic field and 0.14 W cm^−2^ laser compared to the action by the magnetic field alone, and more interestingly, is 30% higher than the sum of the two individual actions. The synergistic benefit of the magneto- and photo-thermal properties of the heteromeric structure can reduce the strengths of the magnetic field and laser intensities as well as their irradiation time to levels lower than those required in their hyperthermia applications individually. *In vitro* cytotoxicity analysis performed on HepG2 liver cancer and Hela cervical cancer cell lines showed that IC_50_ values were 83 ± 5.6 μg mL^−1^ (for HepG2) and 122.6 ± 19.8 μg mL^−1^ (for Hela cells) after 48 h of incubation, therefore, the nanoparticles are moderately cytotoxic and nontoxic to HepG2 and Hela cells, respectively; which offers the potential of safe therapy.

## Abstract 2

In this study, we present an experiment showing that designing multifunctional MnFe_2_O_4_–Ag nanoparticles to act as a dual hyperthermia agent is an efficient route for enhancing their heating ability.

## Graphical abstract

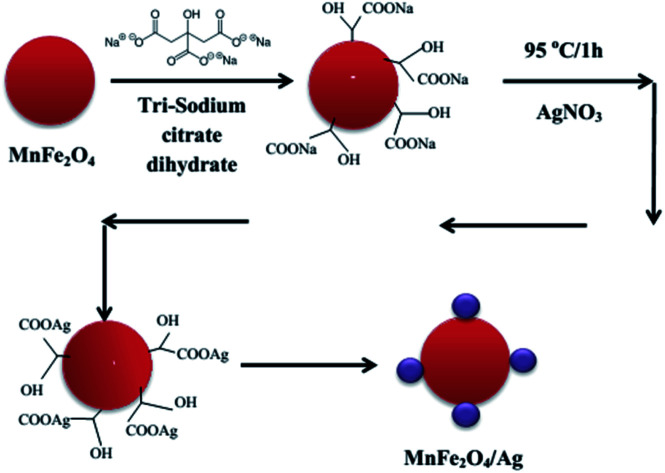

## 1. Introduction

Recent attempts to increase specific absorption rate (SAR) in magnetic nanomaterials used in magnetic fluid hyperthermia (MFH) have led to the discovery of multifunctional hybrid nanoparticles. Hybrid nanostructures with two or more distinctive functional components often have unique advantages that cannot be gained by individual components.^1,2^ To date, several magnetic hybrid carbon nanotubes have been developed for magnetic resonance (MR) imaging and MR-guided photothermal treatment of tumors.^3,4^ Hybrid NPs with core/shell or composite nanostructures made from plasmonic metals (Au, Ag, or Cu) and magnetic nanoparticles (Fe_3_O_4_, γ-Fe_2_O_3_ or MnFe_2_O_4_) are used in imaging diagnostics and hyperthermia therapeutics. In such applications, optical imaging and photothermal are based on the strong localized plasmon resonant optical response of the metallic components of these multifunctional hybrid nanostructures, whereas contrast enhancement for MRI, targeted delivery, and thermal heating can be provided by magnetic components.^5–8^ Regarding the heating properties, using such multifunctional hybrid nanoparticles can benefit synergistically the individual magnetothermal and photothermal efficiencies to improve the safety in tumor treatments *via* reducing strengths of stimulus electromagnetic fields and/or the required amount of the nanoparticles agents. Therefore, this topic has recently attracted a large attention of the scientific community, especially after the discovery of amplified heating efficiency in magnetite nanocubes irradiated simultaneously by an alternating magnetic field (AMF) and a laser.^9^

In the past decade, among plasmonic nanostructures gold nanoparticles (NPs) had most been used as therapeutic agents because of their adjustable plasmon resonance in the near infrared (NIR).^10^ This feature is useful in the treatment of cancer through *in vitro*^11,12^ and *in vivo*^10,13^ photothermal tumor ablation. Hence, if gold nanoparticles and magnetic nanoparticles are used simultaneously, the properties of these nanomaterials can act as therapeutic and diagnostic agents. For example, Balasubramanian *et al.*^14^ demonstrated the application of a magnetic hybrid Au nanocomposite for combined chemo-, photo-, and magnetic hyperthermia therapies against pancreatic and breast cancer. However, the fabrications of such multifunctional gold nanoparticles require multistep and expensive processes. The limited scalability and reproducibility of the process hinder the commercialization of the nanoparticles. Therefore, developing alternative materials with increased reproducibility and reduced costs is significant.

In addition to Au NPs, silver (Ag) NPs are classified as good plasmonic heating agent^11^ along with being as excellent anticancer or antibacterial biological actions, and created new opportunities for the extensive study of using Ag NPs in biomedical fields.^15–17^ Ag NPs can enhance the efficiency of hyperthermia.^18–20^ For instance, Wang *et al.*^21^ demonstrated that Ag NPs improve the thermosensitivity of C6 cells. Ding *et al.*^22^ showed that multifunctional magnetite-silver hybrid NPs with core–shell (Fe_3_O_4_@Ag) or heteromeric (Fe_3_O_4_–Ag) structures exhibit higher biocompatibility with SMMC-7721 and L02 cells than individual Ag NPs. They also studied the influence of a combination of Fe_3_O_4_ with Ag NPs on magnetic hyperthermia and found that Ag NPs enhance cancer cell death both *in vitro* and *in vivo*, although no enhancement in SAR by the hybridization was found. Interestingly, the SAR value of the heteromeric structure is about 10% larger than that of the core shell structure. The above results suggested that fabrication of a nanocomposite-like structure with magnetic NPs separated from Ag NPs might be sufficient to gain a hyperthermia agent for efficient hybrid magneto- and photothermal treatments. As to the best of our knowledge, there has been no report studying on the dual heating modality of an Ag containing hybrid nanostructure.

Manganese ferrite (MnFe_2_O_4_) shows a higher magnetic saturation than several other magnetic materials. Moreover, manganese ferrite is an inexpensive, air-stable, biocompatible, and magnetically recoverable material with a high saturation field^23^ and has thus emerged as a potential candidate material for various biomedical applications, such as MRI contrast agent,^24^ hyperthermia treatment,^25^ and drug delivery.^26^

Multifunctional nanomaterials designed to compose of MnFe_2_O_4_ and inorganic compounds, such as silver or graphene oxide have various purposes, such as increasing antibacterial efficiency, enhancing electrochemical performances, *etc.*^27,28^ However, studies on hyperthermia therapy using MnFe_2_O_4_ silver hybrid NPs are still scarce.

Aiming at improving the heating properties of nanoparticles, we attempted to synthesize heteromeric MnFe_2_O_4_–Ag structure with different mass fractions of Ag NPs for combining magnetic hyperthermia and photothermal techniques. The nanoparticles were prepared with a facile co-precipitation technique, and the morphology, structure, magnetic and optical properties as well as heating capability of the resultant hybrids were systematically investigated for potential applications in cancer hyperthermia treatments. We measured and compared the heating power of MnFe_2_O_4_–Ag NPs in three protocols, *i.e.* (i) magnetic inductive heating (MIH), (ii) photothermal heating (PTH) and (iii) both MIH and PTH modes were applied simultaneously (DUAL). It is showed that the SLP can be maximized at equal mass ratio of MnFe_2_O_4_ and Ag NPs. The *in vitro* biocompatibility of the naked MnFe_2_O_4_–Ag hybrids was evaluated by analyzing the IC_50_ data of the agents against HepG2 liver cancer and Hela cervical cancer cell lines in order to deduce their toxicity level acceptable for the biomedical application. Overall, this study aimed to provide a novel and effective approach to improve hyperthermia treatment efficiency.

## 2. Experimental

### 2.1. Materials

Manganese(ii) chloride tetrahydrate (MnCl_2_·4H_2_O, 98%), iron(iii) chloride hexahydrate (FeCl_3_·6H_2_O, 99%), sodium citrate dihydrate (C_6_H_5_Na_3_O_7_·2H_2_O, 99%), sodium hydroxide (NaOH, 97%), and nitric acid silver(i) salt (AgNO_3_, 99%) were purchased from Merck (Germany). All chemicals were analytically pure. Distilled water was used in synthesizing the samples.

### 2.2. MnFe_2_O_4_ NPs synthesis

Nano-sized MnFe_2_O_4_ powder was synthesized using a co-precipitation technique. The details of the synthesis procedure are the same as that described in the work of Pereira *et al.*^29^ with slight adjustments. 4 mmol of manganese chloride (MnCl_2_·4H_2_O) and 8 mmol of ferric chloride (FeCl_3_·6H_2_O) were dissolved in deionized water and thoroughly mixed. The liquid precipitate was warmed up to 90 °C and afterward 4 mmol NaOH as a precipitating agent was added to beyond solution drop by drop with constant stirring, till pH maintained at 11–12. The mixture kept in that condition for 60 min. After the reaction, the product was cooled naturally to room temperature. Finally, the black precipitate was magnetically separated and washed several times with deionized water and dried under vacuum. The produced crystalline MnFe_2_O_4_ nanoparticles were collectively labeled as S0.

### 2.3. Synthesis of manganese ferrite silver hybrid NPs (MnFe_2_O_4_–Ag)

**Fig. 1:**
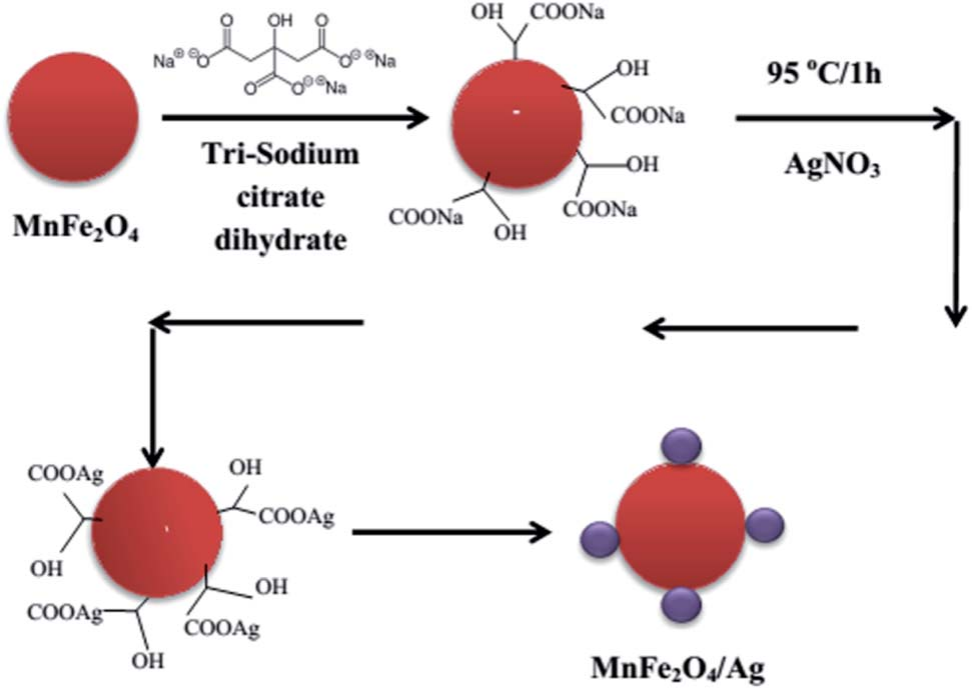
Schematic diagram of synthesizing MnFe_2_O_4_–Ag hybrid NPs.

MnFe_2_O_4_–Ag hybrid NPs were synthesized using the same procedure as described in ref. 30. The schematic diagram of the synthesis steps is illustrated in Fig. 1. First, 35 mL of deionized water and then 20 mg of MnFe_2_O_4_ (obtained in Section 2.2) were added to a beaker and the mixture was stirred well. Then, 2 mL of C_6_H_5_Na_3_O_7_·2H_2_O (0.54 mmol) was added and stirred vigorously at 500 rpm for 10 minutes. The mixture was heated to 95 °C while stirring for 15 minutes. Then, AgNO_3_ was added directly to the solution with 4 various MnFe_2_O_4_ : Ag mass ratio of 2 : 1, 1 : 1, 1 : 2 and 1 : 2.5 for S1, S2, S3 and S4 sample, respectively. The used concentrations of AgNO_3_ and the Ag mass in all the synthesized MnFe_2_O_4_–Ag NPs samples were gathered in Table 1. The mixture was allowed to react for 1 h at a constant temperature of 95 °C while stirring at 500 rpm. After cooling to room temperature, the resulting black precipitate was separated with a magnet, washed for several times with deionized water and dried under vacuum. The final product was used for conducting the characterization.

**Table 1:** Labels of synthesized MnFe_2_O_4_–Ag powder samples

Sample	Mass of MnFe_2_O_4_ (mg)	Concentration of AgNO_3_ (mmol)	Mass of Ag (mg)	Mass ratio
S0	20	0	0	
S1	20	0.09	10	2 : 1
S2	20	0.19	20	1 : 1
S3	20	0.37	40	1 : 2
S4	20	0.46	50	1 : 2.5

### 2.4. Characterization techniques

#### 2.4.1 X-ray diffraction

The crystalline structures of the synthesized powders were analyzed with a Bruker D8-Advance instrument operating at 35 kV and 30 mA, in the reflection mode with Cu Kα line of 1.5406 Å. Data were collected over a 2*θ* range of 20° to 80°, step size 0.02° and time per step of 4 s at room temperature. The detailed structural characterization was analyzed with the Rietveld method.

#### 2.4.2 Raman spectroscopy

All spectra were collected at room temperature using a Jobin-Yvon Lab RAM HR800 Raman Spectrometer with a laser of 830 nm wavelength and power of 17 mW to not affect the sample. The laser beam with its radius of 1.0 μm was focused on the surface of the sample with a 50× objective. The Raman system was calibrated with a silicon semiconductor using the Raman peak at 520 cm^−1^. All spectra were taken in the wavenumber region from 300 to 800 cm^−1^, with a resolution of 0.6 cm^−1^.

#### 2.4.3 Scanning electron microscopy

The morphology of the samples was analyzed using a field emission scanning electron microscopy (FESEM) of Hitachi S-4800. Powder samples were deposited on a Si wafer, dried and inserted in the instrument without further coating. The measurement was performed at energies between 5 and 10 keV. Energy-dispersive X-ray mapping of MnFe_2_O_4_–Ag NPs was obtained using the Hitachi S-4800 FESEM equipped with an energy-dispersive X-ray spectroscopy (EDXS) attachment.

#### 2.4.4 Transmission electron microscopy (TEM)

The morphology and dimensions of the materials were also analyzed in a JEOL JEM-1010 system operating at 120 kV. Particles were measured using ImageJ software and the resulting histograms were constructed by the OriginPro 8.5 (OriginLab, Northampton, MA, USA) software package.

#### 2.4.5 UV-Vis-NIR spectroscopy

Optical absorbance of nanoparticle powders was measured using a Cary 5000 UV-Vis-NIR double beam spectrophotometer (Agilent Technologies, Santa Clara, USA) equipped with an integrating sphere attachment using BaSO_4_ as background, over the 300–800 nm range of wavelength.

#### 2.4.6 Fourier transform infrared spectrometer

Fourier transform infrared (FT-IR) spectra were collected within a wavenumber range of 4000–400 cm^−1^ at a resolution of 1.29 cm^−1^ using a Nicolet 6700 FT-IR spectrometer for the analysis of the bonds of the heteromeric MnFe_2_O_4_–Ag NPs. Specimens were prepared by dilution of MnFe_2_O_4_–Ag powder (2 wt%) in KBr and compressing the mixture into a pellet.

The size distribution of the hydrodynamic diameter of the heteromeric MnFe_2_O_4_–Ag NPs and the stability of the suspension were examined using Zetasizer (Zetasizer Nano, Malvern Instruments, UK). The Zetaseizer system uses the laser diffraction technique to detect the scattered laser light at room temperature. The particles were diluted in water at 10 mg L^−1^ and ultrasonicated (Sonics Vibra Cell, 8 kJ, power 70%, pulse on/off 1 s/1 s). Each sample was measured 5 times to improve the statistics.

The DC magnetic properties of the samples at room temperature were investigated in dry samples using a home-made vibrating sample magnetometer (VSM) under the magnetic field up to 11 kOe.

#### 2.4.7 Magneto- and photo-thermal effect measurements

The induction heating rates of the samples were measured using a commercially available generator UHF-20A combined with laser irradiation (Verdi G SLM-Series). The magneto-optical heating performance of 2 mg mL^−1^ of heteromeric MnFe_2_O_4_–Ag fluids was assessed under simultaneous stimulation by 532 nm continuous laser light irradiation with power density of 0.14 and 0.25 W cm^−2^ and an alternating magnetic field (AMF) at constant frequency of 340 kHz and amplitude range from 100 to 300 Oe for 14 minutes. Temperature of the sample was monitored *in situ* along with the irradiation by using a platinum resistance thermometer (PT 100 sensor).

Efficiency of conversion from the absorbed energy of stimulating field (AMF and/or light) into heat is characterized by specific absorption rate, SAR, which can be experimentally determined using the following equation:^31^1
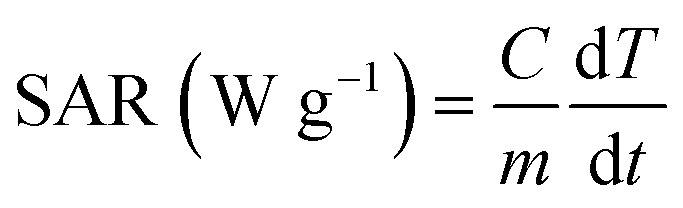
where *C* is the specific heat capacity of water (4.185 J g^−1^ K^−1^), *m* is the concentration (mg mL^−1^ MnFe_2_O_4_–Ag) of the magnetic material in the solution, and d*T*/d*t* is the slope of the measured temperature–time curve. In the current experiments, the temperature slope was calculated *via* analyzing the temperature *versus* time curves for the whole time range, *i.e.* first to fit experimental curves by the following equation:^32^

2 *T* = *T*_p_ + Δ*T*(1 − e^*t*/*t*_m_^)

to gain: Δ*T* – temperature difference between the initial and steady state, and *t*_m_ – the time constant of heating; and then d*T*/d*t* is determined as equal to Δ*T*/*t*_m_.

### 2.5. Cytotoxicity assays

#### 2.5.1 Preparation of stock solutions of drugs

Doxorubicin hydrochloride (Adriamycin®) was provided from Sigma-Aldrich (USA). Stock solutions of 2.5 μg mL^−1^ doxorubicin hydrochloride and further dilutions (0.039, 0.078, 0.156, 0.312, 0.625 and 1.25 μg mL^−1^) were prepared in RPMI1640. The stock solutions were sterilized using 0.22 μ microfilters under laminar flow hood and stored frozen.

#### 2.5.2 Cell line and culture conditions

HepG2 liver cancer and Hela cervical cancer cell lines were assessed, which were procured from the American Type Culture Collection. The cell lines were maintained and propagated in 90% Dulbecco's Modified Eagle's Medium containing 10% fetal bovine serum and 1% penicillin/streptomycin. All media, serum, and antibiotics were provided by Gibco, USA. The cells were cultured as adherent monolayers (*i.e.*, cultured at approximately 80% confluence) and controlled at 37 °C in a humidified 5% CO_2_ atmosphere.

#### 2.5.3 MTS viability assay

*In vitro* cytotoxicity to HepG2 liver cancer and Hela cervical cancer cell were evaluated using MTS assay and were compared with the untreated controls. A total of 180 μL of cells growing in the log phase, were seeded into four 96-well flat-bottom culture plate at a density of 5 × 10^3^ cells per well and subsequently incubated for 24 h. Then, in plate 1 and 2, serial dilutions of doxorubicin (20 μL; final concentration: 0.039–2.5 μg mL^−1^) were added to a final volume of 200 μL and incubated for another 72 h. In plates 3 and 4, serial dilutions of MnFe_2_O_4_–Ag (20 μL) were added. After an incubation period of 48 h, the medium was aspirated and the cells were washed in PBS. Doxorubicin was used as positive controls (40 μL in each well), and the cells treated only with medium were considered as negative controls.

To evaluate cell survival, 20 μL of MTS [3-(4,5-dimethylthiazol-2-yl)-5-(3 carboxymethoxyphenyl)-2-(4-sulfophenyl)-2*H*-tetrazolium] was added to each well (100 μg mL^−1^ in medium) and incubated for 3 h at 37 °C. Colored formazan formed inside the cells was dissolved by adding dimethylsulfoxide to each well, and the optical density of the suspension was measured at 540 nm in a microplate reader (Elisa Spectra MAX Plus 384, USA). The following formula was used to calculate the viability of cell growth: cell viability (%) = (mean of IA value of treated group/mean of IA value of control) × 100.

## 3. Results and discussion

### 3.1. Characteristics of the heteromeric MnFe_2_O_4_–Ag NPs MNPs

**Fig. 2:**
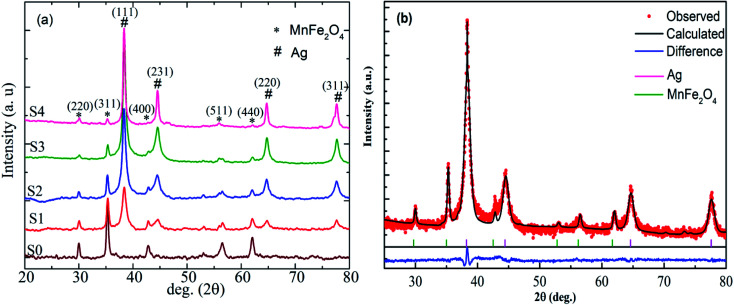
(a) X-ray diffraction patterns of MnFe_2_O_4_–Ag hybrid NPs. (b) Rietveld fitted room temperature *X-ray* diffraction patterns for S2 sample.

The room temperature powder XRD patterns of the Ag-free and various Ag-containing MnFe_2_O_4_ samples are shown in Fig. 2(a). A single cubic phase for pure parent MnFe_2_O_4_ (S0 sample) was observed. The composites showed two different sets of XRD peaks, all of which were successfully indexed with cubic structured MnFe_2_O_4_ and fcc Ag phases. The reflections indexed as (220), (311), (400), (511), and (440) planes (marked with asterisks) confirmed the formation of well-defined single-phase cubic spinel structure characteristics with space group *Fd*3*m*, and the reflections recorded as (111), (200), (231), (220), and (311) planes (marked with hashtags) are attributed to the fcc Ag. No additional peak of any other phase was detected. With increasing Ag content, the intensities of the Ag peaks gradually increased, whereas the intensities of the MnFe_2_O_4_ peaks clearly decreased. Hence, the composites were homogeneous systems composed of cubic MnFe_2_O_4_ and fcc Ag. However, the occurrence of both phases in heteromeric NPs did not affect the crystal structure of each phase, indicating the absence of additional chemical interactions between manganese ferrite and Ag metal phases and the conservation of their symmetry in the composites. To further confirm the coexistence of two phases in the heteromeric MnFe_2_O_4_–Ag NPs, the Rietveld refinements of the S2 sample is exhibited in Fig. 2(b) as an example. The crystallite sizes were calculated using Scherer's equation with diffraction angle using profile of the (311) and (111) peaks for MnFe_2_O_4_ and Ag nanoparticles, respectively. The obtained crystallite size values were listed in Table 2. As can be seen from Table 2, the crystallite size of Ag NPs increases with the increase of Ag concentration, which is opposites with that of MnFe_2_O_4_ nanoparticles.

**Table 2:** The crystallite size of synthesized MnFe_2_O_4_–Ag powder samples

Sample	Crystallite size of MnFe_2_O_4_ (nm)	Crystallite size of Ag (nm)
S0	23.6	0
S1	20.6	4.8
S2	24.0	8.3
S3	18.7	9.8
S4	20.1	16.9

**Fig. 3:**
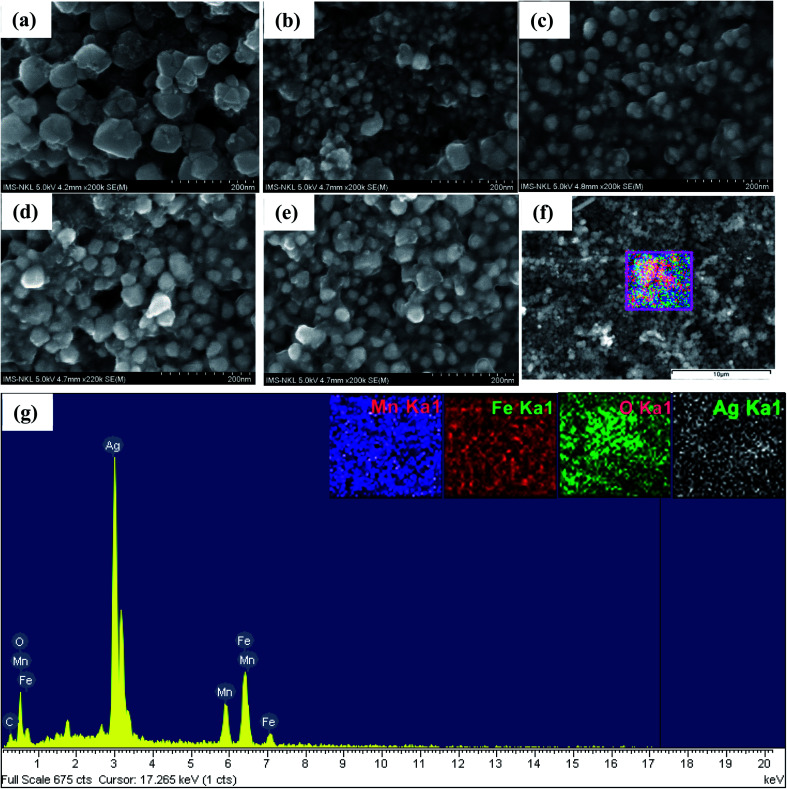
The SEM micrographs of the heteromeric MnFe_2_O_4_–Ag NPs. (a) S0, (b) S1, (c) and (f) S2, (d) S3, (e) S4. (g) The EDS spectrum of the S2 sample, with the inset showing by color the mappings of the four detected elements for the red dotted box in (f) image.

Fig. 3(a)–(e) present the SEM micrographs of the heteromeric MnFe_2_O_4_–Ag NPs at the same magnification. Obviously, except in sample S0, no significant difference in shape was found among particles with different Ag contents. The energy dispersive X-ray (EDS) spectrum analysis results for the red-dotted box region in sample S2 (Fig. 3(f)) is shown in Fig. 3(g). Notably, the peaks in the EDS spectra confirmed the elemental composition (Mn, Fe, Ag, and O), and no other element was detected, verifying that the heteromeric nanoparticles were free of impurity. In combination with the EDX spectra in Fig. 3(g), the corresponding elemental mappings of Mn, Fe, Ag, and O elements are shown in the inset of Fig. 3(g). The elements' map indicated that Mn, Fe, Ag, and O elements were evenly distributed in the heteromeric MnFe_2_O_4_–Ag NPs.

**Fig. 4:**
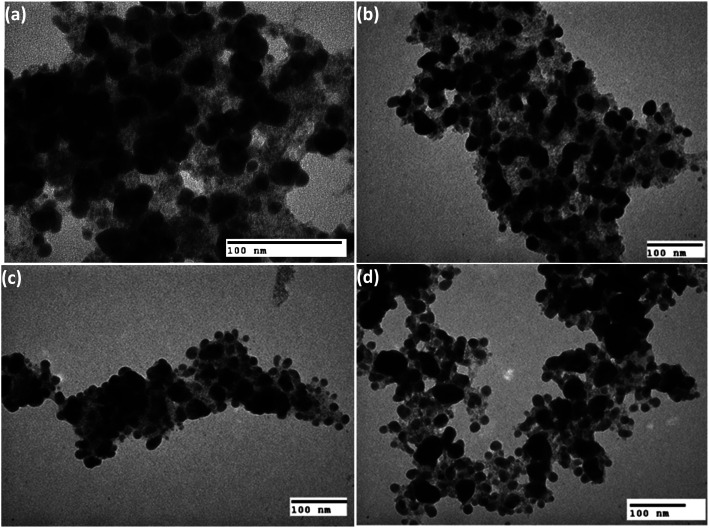
Typical TEM images of the MnFe_2_O_4_–Ag nanoparticles synthesized with (a) 2 : 1, (b) 1 : 1, (c) 1 : 2 and (d) 1 : 2.5 mass ratios of MnFe_2_O_4_ and Ag.

Fig. 4(a)–(d) present the TEM images of the heteromeric MnFe_2_O_4_–Ag NPs synthesized from MnFe_2_O_4_ and Ag metal in weight rations of 2 : 1 (S1), 1 : 1 (S2), 1 : 2 (S3), and 1 : 2.5 (S4). The average particle sizes determined from TEM analysis were 49.8 for the all samples.

**Fig. 5:**
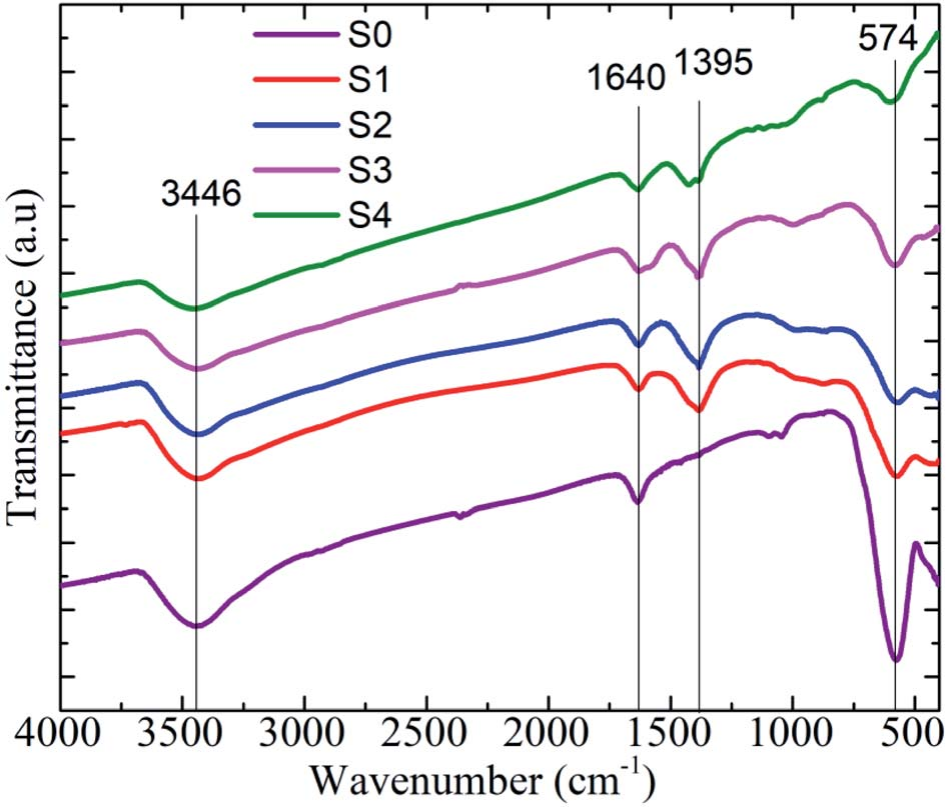
FT-IR spectra of MnFe_2_O_4_–Ag NPs*.*

For characterization of the heteromeric MnFe_2_O_4_–Ag NPs, the FTIR spectrum was recorded in the 400–4000 cm^−1^ range. The results are depicted in Fig. 5. All the samples exhibited strong absorption peaks at 574, 1640, and 3446 cm^−1^. The two broad peaks at 1640 and 3446 cm^−1^ corresponded to the bending and stretching modes of the hydroxyl groups of a small amount of water.^33^ The spectra peak observed at 574 cm^−1^ was the characteristic peak of the intrinsic stretching vibration of iron and oxygen (Fe–O) bond of MnFe_2_O_4_ at the tetrahedral site.^34^ The peak observed at 1395 cm^−1^ represented the COO– asymmetric stretching vibration of COOH in sodium citrate tribasic dehydrate. This observation was in good accordance with the XRD data confirming the phase and typical surface bonding state changes in the samples before and after coating with silver metal.

**Fig. 6:**
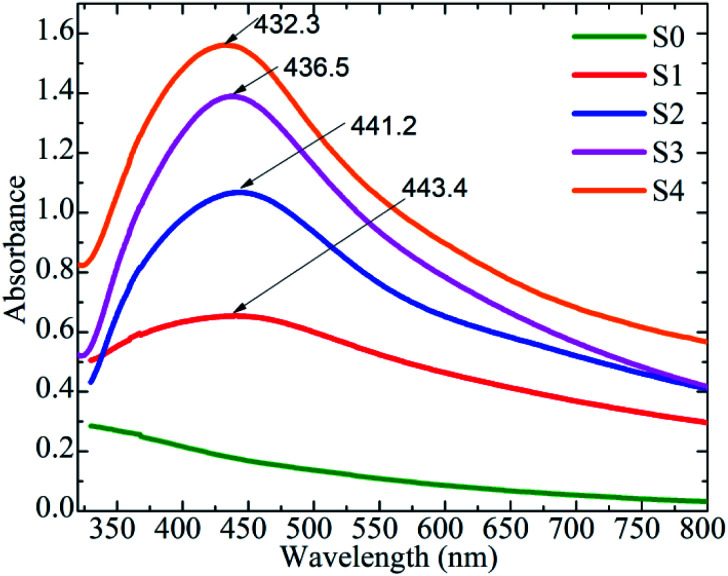
Room temperature UV-Vis absorbance spectrum of MnFe_2_O_4_–Ag hybrid NPs*.*

The localized surface plasmon resonance (LSPR) peak of Ag NPs was identified using the optical property of MnFe_2_O_4_–Ag hybrid NPs at 300–800 nm and UV-Vis absorption spectra. As shown in Fig. 6, except the S0 sample, the samples with various Ag concentrations exhibited the characteristic LSPR peaks of Ag NPs at approximately 432–444 nm. As Ag concentration increased, the LSPR peaks showed slight blue shifts from 444 nm for S1 to 432 nm for S4, because of the local dielectric effect according to the classical Mie theory.^35^ Similar phenomena were reported in literature.^22,36,37^

**Fig. 7:**
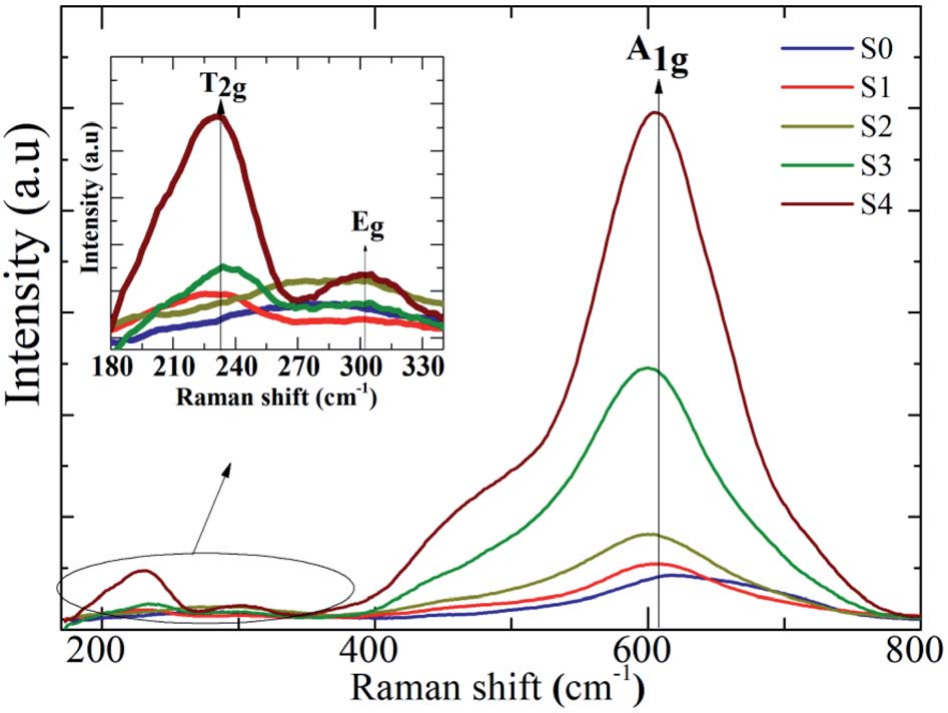
Room temperature Raman spectra of pure and MnFe_2_O_4_–Ag nanoparticles.

The influence of Ag on the Raman scattering of MnFe_2_O_4_ nanoparticles was studied at room temperature through Raman spectroscopy. The spinel ferrite crystal structure MnFe_2_O_4_ has five Raman active modes, namely, A_1g_ + E_g_ + 3T_2g_.^38^Fig. 7 describes the Raman spectra of MnFe_2_O_4_–Ag hybrid NPs at 200–800 cm^−1^. Fig. 7 shows that the Raman spectra of all the samples exhibited three Raman modes, T_2g_, E_g_, and A_1g_, at 220–240, 280–320, and 60–620 cm^−1^, respectively, which closely matched the modes in previously reported data.^39^ In addition, in the heteromeric MnFe_2_O_4_–Ag, the Raman intensities for all bands increased relative to those of pure MnFe_2_O_4_ NPs, and this increase may be attributed to the improvement in electric field induced by the LSPR of the Ag NPs.^40^ The peak intensity significantly increased with Ag content in the MnFe_2_O_4_ NPs.

**Fig. 8:**
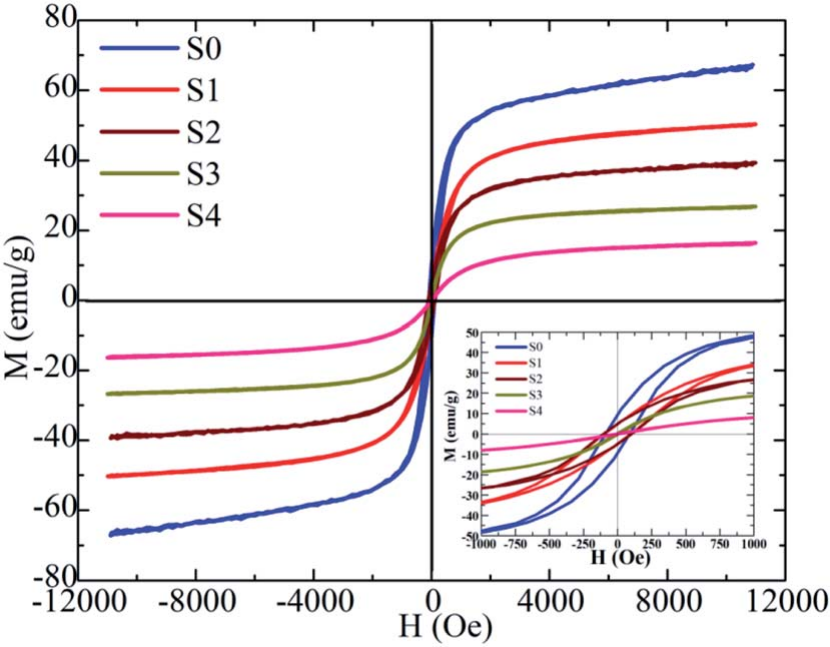
Room temperature magnetization of MnFe_2_O_4_–Ag hybrid NPs.

The magnetic properties of the heteromeric MnFe_2_O_4_–Ag NPs were studied according to their magnetic hysteresis loop at 300 K under an applied magnetic field (*H* = 10 kOe), as shown in Fig. 8. Maximum magnetization decreased from 66.5 emu g^−1^ (for S0 sample) to 16.1 emu g^−1^ (for S4 sample) with increasing Ag concentration because of the increase in the amount of non-magnetic silver. Initially, coercivity (*H*_c_) did not change initially in the S0, S1, and S2 samples but subsequently decreased rapidly with further increase in Ag concentration. The highest values of coercivity (*H*_c_) were 113.5 Oe for S0, S1, and S2 samples and 6.8 Oe for the S4 sample. In general, the coercivity of the magnetic nanoparticles depends on magneto-crystalline anisotropy, microstrain, interparticle interaction, grain size, and shape.^41^ In our study, the *H*_c_ of the heteromeric MnFe_2_O_4_–Ag NPs decreased with increasing Ag content in the S3 and S4 samples. This property can be attributed to the low microstrain of Ag.

### 3.2. Heat generation of the heteromeric MnFe_2_O_4_–Ag NPs

**Fig. 9:**
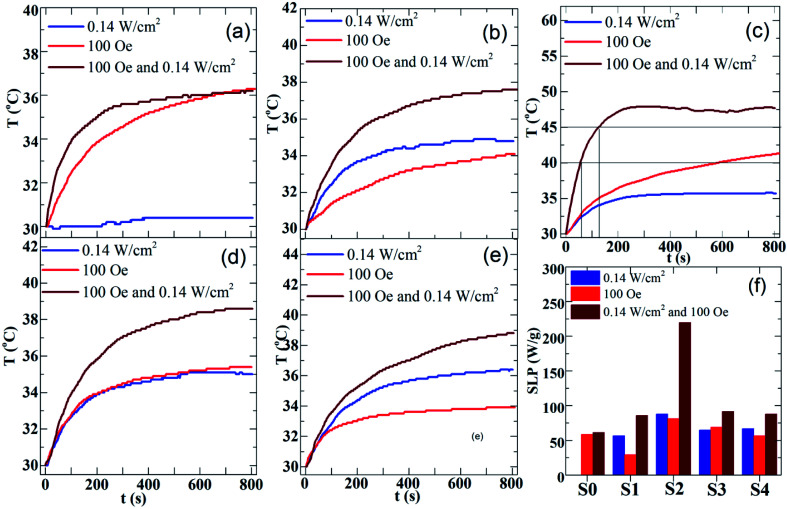
Photothermal heating and magnetic inductive heating curves of the MnFe_2_O_4_–Ag hybrid nanoparticles suspension (2 mg mL^−1^): (a) S0 sample, (b) S1 sample, (c) S2 sample, (d) S3 sample and (e) S4 sample. (f) The field-dependent SAR values of the MnFe_2_O_4_–Ag hybrid nanoparticles suspension.

The magneto- and photo-thermal efficiency of the heteromeric MnFe_2_O_4_–Ag NPs was analyzed in aqueous dispersions, and three modes were used. First, the magnetic fluids were subjected to an alternating magnetic field at 340 kHz and 100 Oe, and magnetic inductive heating (MIH) was evaluated. Then, the hybrid NPs fluids were irradiated with a laser at 532 nm and 0.14 W cm^−2^ for the assessment of photothermal heating (PTH). Finally, both modes of thermal stimulation were applied simultaneously (DUAL, MIH + PTH). Fig. 9(a)–(e) show the time-dependent temperature increase in the MnFe_2_O_4_–Ag fluids with a fixed concentration of 2 mg mL^−1^. In the pure MnFe_2_O_4_ fluid, the temperature increase (Δ*T*) only reached under MIH and DUAL protocols, as shown in Fig. 9(a). In the other samples, temperature increased in the three experimental modes (Fig. 9(b)–(e)). Moreover, the rate of temperature increase was higher when dual-mode stimulation was used compared to the cases of single individual ones. Interestingly, the therapeutic temperature window (42–45 °C) was reached within very short irradiation time, namely less than 2 min for the S2-based fluid. SAR values for the fluids were then calculated based on the Δ*T* and *t*_m_ parameters gained by fitting eqn (2) to the experimental temperature *versus* time curves. The results are presented in Fig. 9(f). The maximum SAR value was obtained in the S2 sample at three measuring conditions. The heating effect is improved obviously, which indicates that a high localized heating can be rapidly achieved by combining a moderate laser irradiation and a low magnetic field, simultaneously. As shown in Fig. 9(f), SAR value of S2 sample under DUAL mode (219.5 W g^−1^) is 2.7 times larger than the one by MIH (81.5 W g^−1^) alone, and more interestingly is about 30% larger than the sum of individual MIH and PTH (87.8 W g^−1^) ones. Another noteworthy finding is that the time, *t*_c_, to reach cancer therapeutic temperature range (40–45 °C) is much shorter for the DUAL mode than for the single modality. As can be seen in Fig. 9(c), the temperature of 42.5 °C is reached after about *t*_c_ = 100 s and 800 s by the synergistic irradiation of 0.14 W cm^−2^ laser light combined with magnetic field of 100 Oe/340 kHz, and only the later action, respectively. This observation for the MnFe_2_O_4_–Ag fluids is quantitatively comparable with the results of the work by Espinosa *et al.* for iron oxide –Au NPs recently reported in ref. 42, where the cancer treatment temperature was achieved in *t*_c_ = 100 s when using simultaneously the laser of 0.3 W cm^−2^ power and alternating magnetic field of 250 Oe/110 kHz. Therefore, the photothermal and magnetothermal properties of MnFe_2_O_4_–Ag nanoparticles show great potential for localized cancer treatments, when considering the advantages in reduction of both strengths of stimulating fields and their irradiation time. In addition, in both PTH and DUAL modes, two regimes were observed, namely, a first linear increase in SAR is observed at low concentrations of Ag, and which tends to have a constant value with increasing Ag concentration, as recently reported.^43–45^ According to the Beer–Lambert law,^46^ the heating transfer of nanoparticles produced by PTH is strongly dependent on nanoparticle concentration, and the light absorption of laser intensity increases with concentration. However, heating capacity reaches a maximum value at a fixed high concentration. This effect can be explained by the following light-to-heat energy transfer equation:

3 *I*_0_ × *S*(1 − 10^−*A*^) × *η* = *m*_sample_ × *C* × d*T*/d*t*

where *I*_0_ (W) is the incident laser power, *S* (cm^2^) is the illuminated area, *A* is the absorbance of the sample at the irradiation wavelength, *η* is the photothermal conversion efficiency from irradiation laser energy to thermal energy, *m*_sample_ (g) is the weight of sample, *C* (J g^−1^ K^−1^) is the specific heat of water, and d*T*/d*t* (°C s^−1^) is the initial slope of the heating curves. In eqn (2), 10^−*A*^ becomes negligible at a high concentration. Therefore, the SAR remained nearly constant with increasing Ag concentration. This result was also obtained using the PTH and DUAL protocols. Conversely, heating generation efficiency for MIH was lower than the values obtained using the PTH and DUAL protocols. Initially, SAR decreased from sample S0 (without Ag) to sample S1 and then reached its maximum value in sample S2. Finally, it gradually decreased in sample S4. Apparently, non-monotonically changes in SAR with decreasing Ag concentration may be caused by another relaxation mechanism apart from the hysteresis loss and Neél–Brownian relaxation, for instance, dipole–dipole interactions, which can be similarly tuned by decreasing magnetic particle volume fraction. A similar property was observed by Martinez-Boubeta *et al.*^47^ Obviously, SAR obtained from the S2 sample in all three conditions reached the highest value. This result implied that an equal mass ratio between MnFe_2_O_4_ and Ag would achieve the optimum experimental SAR value. Therefore, the dependence of SAR on the intensity of the magnetic field and the power of the laser and the evaluation of *in vitro* cytotoxicity should be explored for the purpose of application in cancer therapy.

**Fig. 10:**
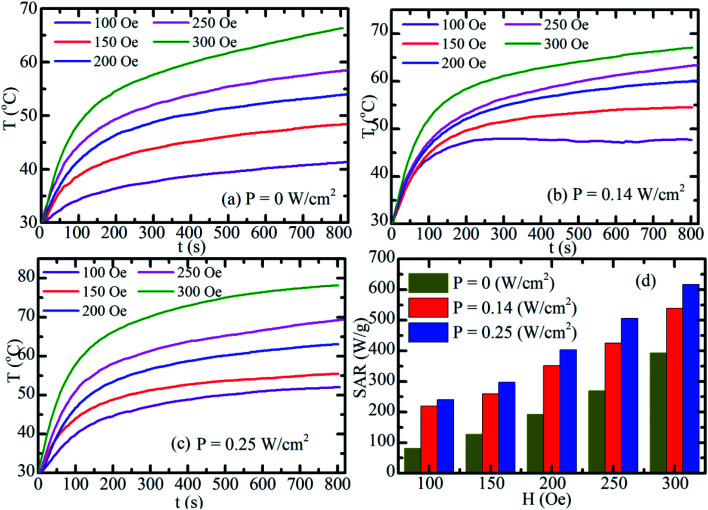
Time dependent temperature curves of S2 hybrid NPs fluid, gained by synergistic (DUAL) stimulations at various amplitudes of magnetic field under 3 different uses of laser light powers: (a) *P* = 0 W cm^−2^, (b) *P* = 0.14 W cm^−2^, (c) *P* = 0.25 W cm^−2^. (d) The field-dependent SAR values of the S2 hybrid NPs fluid.

Fig. 10(a)–(c) show the time-dependent temperature increase of S2-based fluid at 2 mg mL^−1^ concentration. First, the magnetic fluid was subjected to an alternating magnetic field with a fixed frequency of 340 kHz and amplitudes of 100, 150, 200, 250, and 300 Oe for the assessment of MIH alone (Fig. 10(a)). Then, at each of the above magnetic field the temperature increases of the hybrid fluid were conducted under simultaneous irradiation by the laser of power of 0.14 W cm^−2^ (Fig. 10(b)) and 0.25 W cm^−2^ (Fig. 10(c)). As shown in Fig. 10(a)–(c), the heat generated increased with magnetic strength and laser power. The SAR value markedly increased when laser and magnetic stimulation were combined, and this method increased the SAR from 75–250 W g^−1^ with MIH alone to 625 W g^−1^ at the maximum laser power (0.25 W cm^2^; Fig. 10(d)). Magnetic and photo-induced heating can thus be tuned by varying the magnetic field and laser parameters. Espinosa *et al.*^42^ obtained the highest SAR value of 150 W g^−1^ using Fe(16)@Au nanoparticles with MIH/PTH protocols, but the laser power in their study was twofold (0.5 W cm^−2^) that in the current study (0.25 W cm^−2^). Owing to the low concentration (2 mg mL^−1^) of MnFe_2_O_4_–Ag hybrid NPs in our experiment, magnetic hyperthermia combined with photothermal treatment for MnFe_2_O_4_–Ag hybrid NPs optimized the therapeutic condition, as indicated by the SAR. In addition, laser power density (0.25 W cm^−2^) used in this experiment was in the physiologically threshold. A low MnFe_2_O_4_–Ag dose is one of the important advantages in biomedical applications.

### 3.3. Colloidal stability of the heteromeric MnFe_2_O_4_–Ag NPs

**Fig. 11:**
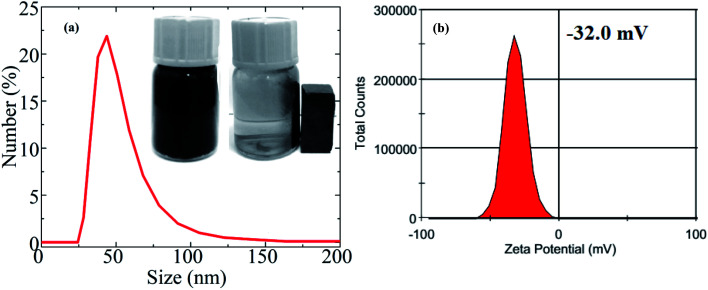
(a) Dynamic light scattering (DLS) size of MnFe_2_O_4_–Ag hybrid NPs in deionized water. The inset shows the dispersion and separation process of MnFe_2_O_4_–Ag hybrid NPs dispersed from deionized water under an external magnetic field. (b) *Z*-Potential of MnFe_2_O_4_–Ag hybrid NPs dispersed in water.

The hydrophilic nature of materials is an important parameter for biomedical applications. Fig. 11(a) represents the mean hydrodynamic size (*D*_H_) of an S2-based fluid. The average hydrodynamic size (*D*_H_) obtained by DLS technique was ∼48 nm, whereas the average core size estimated from TEM was 37.2 nm. The *D*_H_ was larger than the core size obtained from TEM because the *D*_H_ included the shell of the surfactant and dissolved molecules around the NPs. The *D*_H_ in this study was significantly less than 250 nm, so the fluid is quite suitable for biomedical applications. The MnFe_2_O_4_–Ag hybrid NPs were well-dispersed and colloidally stable in aqueous solutions and showed no sign of physical agglomeration or aggregate formation for at least 3 weeks (inset in Fig. 11(a)). The highly hydrophilic property of the MnFe_2_O_4_–Ag hybrid NPs was mainly attributed to the existing of OH-group in the sample. Moreover, zeta potential was measured and used to determine the long-term colloidal stability of MNPs in aqueous solutions. The result is shown in Fig. 11(b). The high negative charge (−32 mV) of the surface implied increase in electrical charge on the surface of the NPs, causing electrostatic repulsion between particles. This result indicated that the long hydrophilic chains of the OH-group played an important role in dispersing the NPs. Moreover, the suspended MnFe_2_O_4_–Ag hybrid NPs were easily collected by a magnet, as shown in the inset in Fig. 11(a), suggesting that they can be controlled by an external magnetic field.

### 3.4. *In vitro* cytotoxicity assay of the heteromeric MnFe_2_O_4_–Ag NPs

**Fig. 12:**
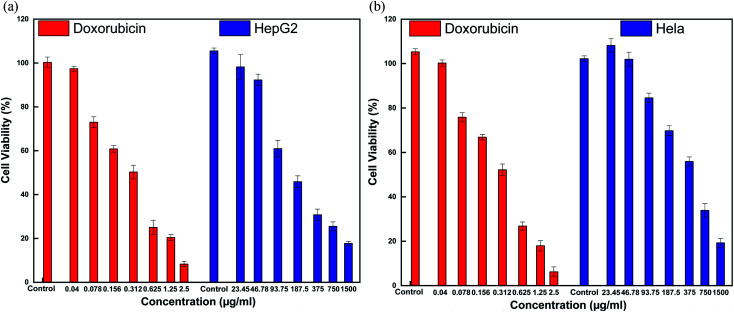
Cytotoxicity of MnFe_2_O_4_–Ag hybrid NPs and Doxorubicin after 48 h exposures determined by the MST assay in HepG2 cells (a) and HeLa cells (b).

In general, assessing the biocompatibility of heteromeric MnFe_2_O_4_–Ag NPs using cytotoxicity assay is important. The cytotoxicity of the S2 sample with various MnFe_2_O_4_–Ag concentrations was evaluated against HepG2 liver cancer and Hela cervical cancer cell lines with a standard MTS assay. The MTS assay of the HepG2 cells and Hela cells after 48 h of contact with 23.45, 46.9, 93.75, 187.5, 375, 750, and 1500 μg mL^−1^ of MnFe_2_O_4_–Ag NPs. The results obtained from these studies are depicted in Fig. 12. Cell viability was found to be concentration dependent, and with the increasing concentration of the S2 sample, cell viability decreased. After the concentration of the MnFe_2_O_4_–Ag NPs increased from 187.5 μg mL^−1^ to 1500 μg mL^−1^, cell viability percentage was reduced. After 48 h of incubation, control treatments showed the highest cell viability percentage (>100%), and the lowest (<19%) was observed at 1500 μg mL^−1^ nanoparticle concentration. IC_50_ values of 83 ± 5.6 μg mL^−1^ (*R*^2^ = 0.98) and 122.6 ± 19.8 μg mL^−1^ (*R*^2^ = 0.97) were observed for HepG2 and Hela cells after 48 h of incubation, respectively. In contrast, doxorubicin (positive control) in the tested concentrations of 2.5 μg mL^−1^ killed most of the cells. IC_50_ values were 0.23 ± 0.07 μg mL^−1^ (*R*^2^ = 0.98) and 0.25 ± 0.05 μg mL^−1^ (*R*^2^ = 0.97) for HepG2 and Hela cells, respectively, evidencing toxic effect on these cells of doxorubicin. Similarly, cytotoxic activity against N27 dopaminergic neuronal cells of Mn nanoparticles was induced in time- and dose-dependent manners was reported in the literature.^48^

**Fig. 13:**
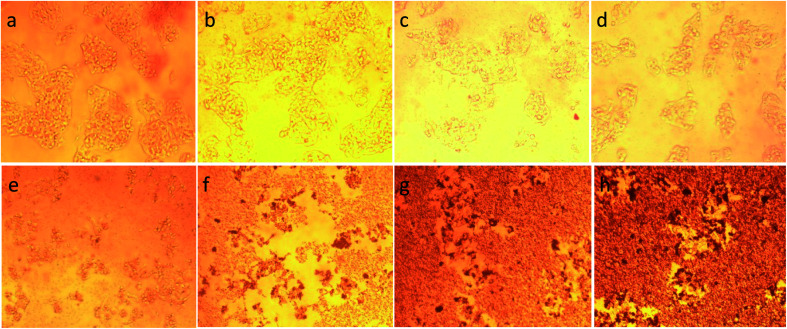
Optical micrograph of HepG2 cells after 48 h incubation with different concentrations of MnFe_2_O_4_–Ag hybrid NPs*.* (a) Control cells, (b) 23.43 μg mL^−1^, (c) 46.87 μg mL^−1^, (d) 93.75 μg mL^−1^, (e) 187.5 μg mL^−1^, (f) 375 μg mL^−1^, (g) 750 μg mL^−1^, and (h) 1500 μg mL^−1^.

**Fig. 14:**
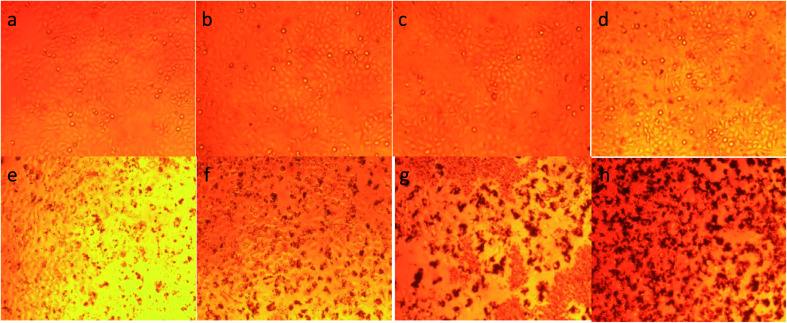
Optical images of Hela cells after 48 h incubation with different concentrations of MnFe_2_O_4_–Ag hybrid NPs*.* (a) Control cells, (b) 23.43 μg mL^−1^, (c) 46.87 μg mL^−1^, (d) 93.75 μg mL^−1^, (e) 187.5 μg mL^−1^, (f) 375 μg mL^−1^, (g) 750 μg mL^−1^ and (h) 1500 μg mL^−1^.

**Fig. 15:**
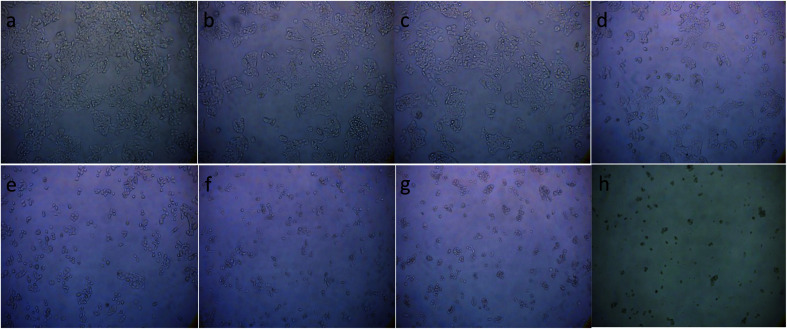
Optical micrograph of HepG2 cells after 48 h incubation with different concentrations of doxorubicin. (a) Control cells, (b) 004 μg mL^−1^, (c) 0.078 μg mL^−1^, (d) 0.156 μg mL^−1^, (e) 0.312 μg mL^−1^, (f) 0.625 μg mL^−1^, (g) 1.25 μg mL^−1^, and (h) 2.5 μg mL^−1^.

**Fig. 16:**
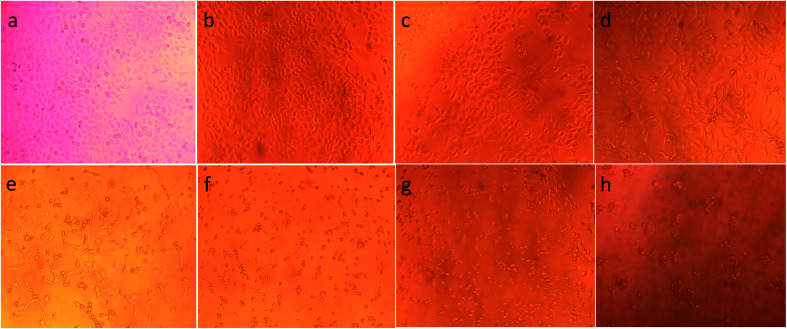
Optical micrograph of Hela cells after 48 h incubation with different concentrations of doxorubicin. (a) Control cells, (b) 004 μg mL^−1^, (c) 0.078 μg mL^−1^, (d) 0.156 μg mL^−1^, (e) 0.312 μg mL^−1^, (f) 0.625 μg mL^−1^, (g) 1.25 μg mL^−1^, and (h) 2.5 μg mL^−1^.

To illustrate the progress of the experiment on the cytotoxic activity, we presented optical micrographs in Fig. 13–16. Two control samples had nearly the same viability percentage in the HepG2 cells and Hela cells. Both types of cells survived at least not shorter than 48 h. When the cells were exposed to higher concentrations of MnFe_2_O_4_–Ag NPs, they showed progressive increases in apoptotic and necrotic activity. At 750 μg mL^−1^, the cells were difficult to observe (see Fig. 13(g), (h) and 14(g), (h)) after 48 h of incubation. The high concentrations of MnFe_2_O_4_–Ag NPs increased the apoptotic and necrotic activities in the HepG2 cells to a higher degree than in the Hela cells. The mechanism of toxicity of magnetic nanoparticles was discussed in detail in literatures. The studies showed that magnetic nanoparticles of high concentrations induce cell necrosis.^49,50^ Most authors proposed that the mechanism of necrosis inducing is arisen from the generation of reactive oxygen species (ROS).^51^ High ROS levels can damage cells membranes by lipid peroxidation, interrupting DNA, modulating gene transcription, modifying proteins and consequence in decline in physiological function and cell death. However, the high IC_50_ indicate that MnFe_2_O_4_–Ag based-fluid is totally nontoxic to the Hela cells and approximately nontoxic to the HepG2 cells. With such toxicity behavior of the naked MnFe_2_O_4_–Ag nanostructure, we believe the fabricated nanohybrid is quite safe in cancer hyperthermia application.

## 4. Conclusions

In this work, the magnetic/plasmonic nanoparticles of heteromeric MnFe_2_O_4_–Ag structure were fabricated and thoroughly characterized, and their biocompatibility with HepG2 and Hela cells was demonstrated. Through XRD study, it was confirmed that formation of hybrided Ag NPs did not change the crystal structure of MnFe_2_O_4_ nanoparticles, and the enhancement of Raman spectrum was clarified to be due to the localized surface plasmon resonance. The magnetization at room temperature decreases with the silver content, with the maximum of 65.5 emu g^−1^ obtained. The synthesized MnFe_2_O_4_–Ag displayed excellent chemical stability, colloid stability, photostability and biocompatibility. Furthermore, multifunctional MnFe_2_O_4_–Ag is able produce an extra increment of the heating effect in the simultaneous irradiation of magnetic field and laser, which makes the synergistic effect of magnetically induced heating and photothermal heating of 30% stronger than the sum of the two individual ones. The highest SAR was achieved to be 625 W g^−1^ for MnFe_2_O_4_/Ag with equal mass ratio at combined magnetic field of 340 kHz and 300 Oe and laser at a power density of 0.25 W cm^−2^. In particular, the role of combining photothermia with magnetic hyperthermia was addressed. Our results imply that the combination of magnetic and plasmonic properties in the nanohybrids directly and strongly influence the heat generation and, therefore, lead to large SAR in MnFe_2_O_4_/Ag nanofluids. Moreover, the therapeutic temperature can rapidly reach with interval during lower than for classical magnetic hyperthermia treatment. The features of much higher SAR and lower *t*_c_ are particularly important for cancer treatment applications as providing possibility to lower the NPs concentration, and/or lower the strengths as well as shorten the irradiation time of the magnetic and optical stimulating sources. Additionally, bare nanofluid is non-toxic to cells thus it is able to apply them in hyperthermia cancer treatment.

## Conflicts of interest

The authors declare that they have no known competing financial interests or personal relationships that could have appeared to influence the work reported in this paper.

## Supplementary Material
