# Correction: Sensitive MnFe_2_O_4_–Ag hybrid nanoparticles with photothermal and magnetothermal properties for hyperthermia applications

**DOI:** 10.1039/d1ra90155a

**Published:** 2021-10-12

**Authors:** T. T. N. Nha, P. H. Nam, N. X. Phuc, V. Q. Nguyen, N. H. Nam, D. H. Manh, L. T. Tam, N. T. N. Linh, B. T. V. Khanh, L. T. Lu, L. H. Nguyen, P. T. Phong

**Affiliations:** Graduate University of Science and Technology, Vietnam Academy of Science and Technology 18 Hoang Quoc Viet Hanoi Vietnam; Institute of Materials Science, Vietnam Academy of Science and Technology 18 Hoang Quoc Viet Hanoi Vietnam namph.ims@gmail.com; Duy Tan University 03 Quang Trung Da Nang Vietnam; University of Science and Technology of Hanoi (USTH), Vietnam Academy of Science and Technology 18 Hoang Quoc Viet, Cau Giay Hanoi Vietnam; Vinh University 182 Le Duan Vinh Vietnam; Thai Nguyen University of Sciences Tan Thanh Ward Thai Nguyen City Vietnam; Faculty of Biology, VNU University of Science, Vietnam National University Vietnam; Institute for Tropical Technology, Vietnam Academy of Science and Technology 18 Hoang Quoc Viet Hanoi Vietnam; Laboratory of Magnetism and Magnetic Materials, Advanced Institute of Materials Science, Ton Duc Thang University Ho Chi Minh City Vietnam; Faculty of Applied Sciences, Ton Duc Thang University Ho Chi Minh City Vietnam; University of Management and Technology Ho Chi Minh City Vietnam pham.phong@umt.edu.vn

## Abstract

Correction for ‘Sensitive MnFe_2_O_4_–Ag hybrid nanoparticles with photothermal and magnetothermal properties for hyperthermia applications’ by T. T. N. Nha *et al.*, *RSC Adv.*, 2021, **11**, 30054–30068. DOI: 10.1039/D1RA03216J

The authors regret that an incorrect grant number was shown in the acknowledgements section of the published article. The corrected section should read:

This work was supported by the Vietnam Academy of Science and Technology (VAST) by Program of Development in the field of Physics by 2020 under grant number KHCBVL.02/20-21, and partly by AOARD Award No. FA2386-17-1-4042.

The Royal Society of Chemistry apologises for these errors and any consequent inconvenience to authors and readers.

## Supplementary Material

